# Mitochondrial Transport in Bone Metabolism Homeostasis: Molecular Mechanisms and Targeted Therapeutic Strategies

**DOI:** 10.3390/ijms27125193

**Published:** 2026-06-08

**Authors:** Xiuyuan Wang, Jianying Liu, Haochuan You, Yixiang Zhao, Hongyin Fu, Zhongcheng Liu, Yong Sun, Bin Geng, Xingwen Wang, Yayi Xia

**Affiliations:** Gansu Key Laboratory of Orthopaedics, Department of Orthopaedics, Lanzhou University Second Hospital, Lanzhou 730000, China; wangxiuyuan101@163.com (X.W.); 320220905300@lzu.edu.cn (J.L.); youhaochuan33@163.com (H.Y.); zyzhaoyx@163.com (Y.Z.); fuhy2024@lzu.edu.cn (H.F.); liuzhch_14@163.com (Z.L.); suny2024@lzu.edu.cn (Y.S.); cxxxf@foxmail.com (B.G.)

**Keywords:** mitochondrial transport, intercellular communication, bone metabolism, metabolic bone diseases, targeted therapy, bone homeostasis

## Abstract

The homeostasis of bone metabolism relies on the precise synergistic regulation among osteoblasts, osteoclasts, and osteocytes. Disruption of this regulatory network underlies the pathogenesis of osteoporosis, osteonecrosis, osteoarthritis, and other metabolic bone diseases. As an emerging mode of intercellular communication, mitochondrial transport delivers functional mitochondria to impaired cells, thereby reshaping cellular metabolism, alleviating oxidative stress, and restoring cell function. It thus plays an irreplaceable role in maintaining bone metabolic homeostasis. However, studies focusing on mitochondrial transport in bone metabolism are lacking, and the underlying molecular mechanisms have not been elucidated. For metabolic bone diseases, many bottlenecks exist in clinical translation. This review comprehensively summarizes the core molecular mechanism involved in mitochondrial transport, its regulatory functions in bone metabolism homeostasis, its association with metabolic bone diseases, and intervention strategies targeting mitochondrial transport. We aim to provide novel insights into the mechanism and targeted therapy of metabolic bone diseases.

## 1. Introduction

Bone, as an important supportive and metabolic organ of the human body, maintains its structural integrity and functional stability through the dynamic balance between bone formation and bone resorption, namely, bone remodeling [1]. The functional network composed of osteoblasts, osteoclasts and osteocytes synergistically regulates the process of bone remodeling. Disruptions in any component of this regulatory network can precipitate metabolic bone disorders—such as osteoporosis, osteonecrosis, and osteoarthritis—which represent a significant global public health burden, particularly among aging populations [2,3]. Although the existing studies have analyzed the mechanism of bone metabolism imbalance from the perspectives of hormone regulation, signaling pathways and cell senescence, there is still a lack of efficient and safe intervention targets in clinical practice, which indicates that key scientific problems related to the network regulating bone metabolism have not been explored.

Mitochondria are the energy factory of eukaryotic cells and are involved in various biological processes, such as calcium signal transduction and oxidative stress. Mitochondria are the core organelles that maintain cell function [4]. Recent studies have confirmed that mitochondria are not static in cells but can be actively transported and transmitted between cells via mitochondrial transport [5]. This process can result in the delivery of functional mitochondria to damaged or metabolically defective cells, the restoration of energy generation, the elimination of oxidative stress, and the rebuilding of cell function. It is an important defense mechanism through which tissues resist damage and maintain homeostasis. Mitochondrial transport includes mainly tunnelling nanotubes (TNTs), extracellular vesicles (EVs), gap junction channels (GJCs), cell fusion, and free mitochondrial release and capture. It is precisely regulated by the MIRO1/2-TRAK1/2 adaptor complex, motor proteins, the cytoskeleton, calcium signals, energy states, and stress signals [6,7,8,9]. Many studies have confirmed that mitochondrial transport is also important for the regulation of bone metabolic homeostasis, providing a new perspective for the study of bone metabolic diseases [10,11,12,13].

In recent years, an increasing number of researchers have focused on the relationship between mitochondrial function regulation and bone metabolism. There have been abundant reviews on the relationships among mitochondrial fission, fusion and bone metabolism. However, few reviews on the regulation of bone metabolism homeostasis by intercellular mitochondrial transport exist. It is difficult to comprehensively review the research progress, core bottlenecks and development directions in this field [14,15,16,17]. On the basis of these findings and the latest research results, this review comprehensively explores the core molecular mechanism of mitochondrial transport and its role in regulating osteocyte function. This study also examines the intrinsic relationship between mitochondrial transport abnormalities and bone metabolic diseases, and summarizes the potential intervention strategies and clinical applications of targeting mitochondrial transport. These results will provide new directions for pathogenesis research and the identification of new therapeutic drug targets and fill the gaps in bone metabolic disease research to advance the field.

## 2. Concept and Classical Pathway of Mitochondrial Transport

### 2.1. Definition, Biological Characteristics and Physiological Significance of Mitochondrial Transport

**Figure 1:**
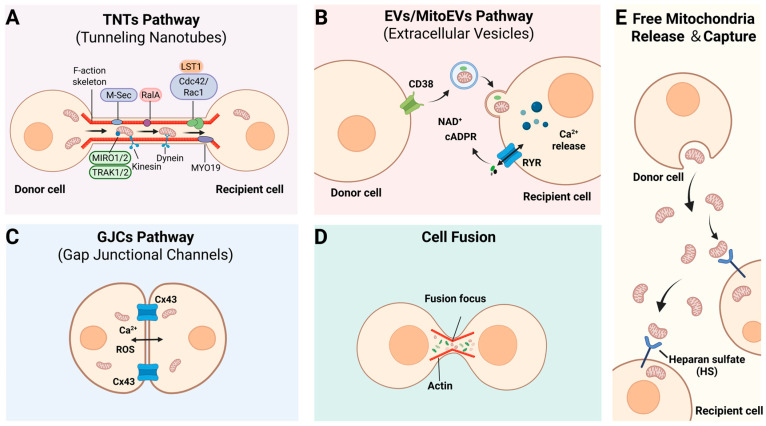
Major routes and molecular mechanisms of intercellular mitochondrial transfer. Intercellular mitochondrial transfer is primarily mediated via five pathways, which collectively constitute a comprehensive transcellular mitochondrial transport network: (**A**) Tunnelling nanotubes (TNTs): TNTs represent the primary and most efficient contact-dependent pathway. Their formation is synergistically regulated by M-Sec, the exocyst complex, and small GTPases. The directional movement of mitochondria within TNTs is highly dependent on the MIRO1/2-TRAK1/2 adaptor protein complex and motor proteins. (**B**) Extracellular vesicles (EVs): EVs are a contact-independent, long-range regulatory pathway that achieves transfer through the secretion of vesicles containing mitochondrial components. The NAD+/CD38/cADPR/Ca2+ signaling pathway serves as the core mechanism for EV-mediated transcellular mitochondrial transfer. (**C**) Gap junction channels (GJCs): GJCs rely on connexins (e.g., Cx43) to mediate the exchange of substances and mitochondrial transfer between adjacent cells. (**D**) Cell fusion: Cell fusion involves the direct fusion of plasma membranes to facilitate the exchange of organelles, including mitochondria. (**E**) Release and capture of free mitochondria: Free mitochondria released under stress conditions are specifically captured by recipient cells. Heparan sulfate (HS) may play a crucial role in the capture process of free mitochondria. Created in BioRender. Wang, X. (2026) https://BioRender.com/dzw9xwf.

Mitochondrial transport, also known as mitochondrial transmission and horizontal mitochondrial transfer, refers to the process in which donor cells deliver intact functional mitochondria, mitochondrial vesicles or mitochondrial components to recipient cells through specific pathways. Under physiological or stress conditions, these transferred elements undergo fusion, localization, functional activation or degradation within recipient cells [18]. This process has a high degree of temporo-spatial specificity and functional targeting; it can quickly respond to external stimuli such as energy deficiency, oxidative stress, hypoxia, and mechanical damage and achieve complementarity and synergy between intercellular metabolic states [19]. From a biological function perspective, the core importance of mitochondrial transport between cells is reflected in three aspects: first, to supplement damaged cells with functional mitochondria, quickly restore ATP production, and ensure cell survival; second, to remove excessive reactive oxygen species in recipient cells, alleviate oxidative stress damage, and maintain redox balance; and third, to transmit mitochondrial DNA and metabolic signals, reshape the metabolic phenotype of recipient cells, and regulate cell differentiation, apoptosis and the inflammatory response [20]. In the bone tissue microenvironment, this process becomes an important metabolic communication link between osteoblasts, osteoclasts and osteocytes and participates in the dynamic regulation of bone remodeling [21]. Studies have shown that mitochondria derived from bone marrow mesenchymal stem cells can be transferred to functionally impaired osteoblasts through exosomes to restore their oxidative phosphorylation activity and mineralization ability. Mitochondria released by osteoblasts can be internalized by osteoclasts and inhibit excessive bone resorption by regulating intracellular ROS levels [22,23] (Figure 1).

### 2.2. Mitochondrial Transport Pathways

Currently, mitochondrial transport is achieved mainly through TNTs, EVs, GJCs, cell fusion and free mitochondrial release and capture. It has its own structural dependence, transmission efficiency, action distance and regulation mode, which together constitute a complete cross-cell mitochondrial transmission network.

#### 2.2.1. TNTs

TNTs are the most important and most efficient contact-dependent pathway mediating mitochondrial transport. TNTs are long, thin, membrane-like tubular structures formed by F-actin as a skeleton across the intercellular space. The diameter of TNTs is usually between 50 and 200 nm. TNTs can form stable physical connections between cells, allowing mitochondria, vesicles, calcium ions and signaling molecules to be directly transferred [24]. TNTs are particularly important in the highly networked anatomical structure of osteocytes. Through dendritic extensions, osteocytes can form many TNT connections, thereby achieving rapid distribution and replenishment of mitochondria in the cell network [11].

The formation of TNTs may involve multiple mechanisms. The actin-driven protrusion model suggests that TNTs begin with a long protrusion of a cell extending in the direction of the donor cell. This process is synergistically regulated by cytoskeleton regulators: M-Sec interacts with RalA GTPase and exosome complexes to initiate membrane protrusion formation and actin remodeling. In addition, small Rho GTPases (such as Cdc42 and Rac1) can finely regulate actin polymerization during TNT formation [25]. Another mechanism related to TNT formation is the cell displacement model, also known as the filamentous pseudopod bridge model. The model proposes that two cells promote the extension of the membrane tube during separation from each other, accompanied by the polymerization of actin in the lumen [26]. The above two mechanisms may be synergistic on the basis of the mechanical microenvironment and the interaction between adhesion and cytoskeleton dynamics. The recently proposed tip junction mechanism further explains the process of closed-end TNT formation, which supports bidirectional ion transport between cells. Such TNTs originate from a double pseudopod bridging structure composed of two filamentous pseudopods. Through the accumulation of mechanical energy, filamentous pseudopods are twisted, and TNTs with closed ends are ultimately formed through interactions with intercellular cadherins [27].

The formation of TNTs involves several key molecules, including exosome complexes, M-Sec (tumor necrosis factor-α-induced protein 2), small GTPases, and leukocyte-specific transcript 1 (LST1). Exosome complexes are responsible for mediating the fusion of Golgi-derived secretory vesicles with the plasma membrane during exocytosis and are widely involved in the morphological remodeling of the cell membrane, such as the formation of neurites, cilia, filopodia and extracellular vesicles [28,29,30]. This complex interacts with various small GTPases, such as Rho1, Rho3, Cdc42 and RalA, to promote actin cytoskeleton remodeling [31,32]. In addition, the exosome complex is involved in the production of TNTs, and this process is regulated by the M-Sec protein [33,34]. M-Sec can promote the assembly of exosome complexes and interact with the complexes and RalA (or Cdc42) to drive actin cytoskeleton remodeling, which is key for TNT formation [25]. In the initial stage of TNT formation, M-Sec directly binds to phosphatidylinositol (4,5)-diphosphate through its N-terminal polybasic region, thereby anchoring to the cell membrane. The binding of M-Sec to RalA depends on the positive charge on the C-terminal surface [35]. Filopodia transformation is also considered one of the potential mechanisms of TNT formation: the effector protein RalBP1 of Cdc42 can regulate actin recombination during filopodia formation. LST1, a transmembrane protein, can recruit RalA to the bottom of the cell membrane, increase the interaction between RalA and exosome complexes, and recruit actin cross-linked protein filament protein [36,37]. Under the synergistic effect of LST1, M-Sec, RalA and exosome complexes, actin filaments can be remodeled and cross-linked, eventually triggering cell membrane protrusions and fusion, thus completing the construction of TNTs [38]. The formation and structural stability of TNTs depend on M-Sec, Cx43, actin regulatory protein and other molecules, whereas the directional movement of mitochondria in TNTs depends on the synergistic effect of the MIRO1/2-TRAK1/2 complex and motor protein, which is a key mitochondrial transmission mechanism in response to mechanical stress and injury stimulation in the bone microenvironment [39,40].

#### 2.2.2. GJCs

Gap junction channels (GJCs) are specialized intercellular junction structures that can establish direct cytoplasmic connectivity between adjacent cells and realize the exchange of ions, metabolites and small signal molecules. Gap junction proteins are a protein family widely distributed in a variety of cells. Six rod-shaped connexin subunits can be assembled on the plasma membrane to form a connector (also known as a half-channel), with a nanoscale hydrophilic pore in the center. As a precursor structure of gap junctions, the half channel can also function independently to form a channel connecting the internal and external environment of the cell [41,42]. The two half-channels are docked in a head-to-head manner, which constitutes a gap junction channel, thereby directly connecting the cytoplasm of two adjacent cells. Gap junction channels allow ions (especially calcium ions) and small molecules to exchange bidirectionally between cells, thereby mediating the transmission of chemical signals or electrical signals [43]. These channels not only provide nutritional support for surrounding cells and enhance the ability of cells to resist damage but also participate in the clathrin-dependent endocytosis of extracellular vesicles [44,45]. Among them, connexin 43 (cx43) plays a central role in mitochondrial transfer. Cx43 is a connexin that has been extensively studied and has been shown to mediate mitochondrial transcellular metastasis. Under resting conditions, hemichannels remain closed to prevent cytoplasmic leakage. During ischemia, injury or Ca^2+^ imbalance, phosphorylation events such as the Cx43 Tyr265 modification will trigger hemichannels on adjacent cells to dock and form functional GJCs, thereby achieving metabolic exchange [46,47]. In a spinal cord injury (SCI) model, MSCs rely on Cx43-dependent GJCs to transmit mitochondria to damaged motor neurons and promote neuronal recovery [48]. In an acute lung injury model, MSCs attach to the alveolar epithelium through Cx43-mediated connections, releasing mitochondria-containing microvesicles to restore ATP levels. Cx43 mutations or mitochondrial dysfunction in MSCs eliminates this therapeutic effect and highlights the important role of complete GJC signal transduction [49].

#### 2.2.3. EVs

EVs constitute a noncontact and remotely regulated transmission pathway. Cells can secrete vesicles containing mitochondrial proteins and mitochondrial DNA and even complete mitochondrial structure under physiological or stress conditions; these vesicles are collectively referred to as mitochondrial vesicles (MEVs) [50]. MEVs reach distal receptor cells through extracellular space diffusion and are taken up by endocytosis, membrane fusion, and other mechanisms and release their mitochondrial content [51]. Unlike TNTs, EV-mediated transport does not depend on direct cell contact and can achieve remote metabolic regulation in the local microenvironment, which plays an important role in the paracrine regulation of osteoblasts and the maintenance of bone marrow microenvironment homeostasis. Moreover, MEVs can also be used as signal carriers, participating in the regulation of inflammation related to bone metabolism and the activation of stem cell function, which is an important supplementary pathway for intercellular mitochondrial transport [52,53,54]. The NAD^+^/CD38/cADPR/Ca^2+^ signaling pathway is the core link of extracellular vesicle (EV)-mediated mitochondrial transcellular transfer. As a transmembrane protein widely expressed on various cell membranes, CD38 can catalyze the formation of cyclic ADP-ribose (cADPR) using nicotinamide adenine dinucleotide (NAD^+^) as a substrate. As a second messenger, cADPR can mediate the release of intracellular calcium stores; it promotes the release of Ca^2+^ stored in the endoplasmic reticulum to the cytoplasm by acting on ryanodine receptors (RyRs, calcium channels) on the endoplasmic reticulum, resulting in a transient increase in the concentration of Ca^2+^ in the cytoplasm, which in turn triggers downstream cell responses [55,56].

#### 2.2.4. Cell Fusion

Cell fusion refers to the plasma membrane fusion of two independent cells, which leads to the sharing of the two cells’ organelles and cytoplasmic compounds while maintaining the integrity of their nuclei. The cells formed by permanent cell fusion share a cytoplasm and have unique karyotypes. Partial cell fusion allows transient but direct intercellular communication, allowing the exchange of multiple protein complexes and even subcellular organelles such as mitochondria [57,58]. Mitochondrial transfer mediated by cell fusion is a precisely regulated multilevel process [59]. Its core molecular mechanism begins with the activation of the G protein-coupled receptor (GPCR)-Ras-mitogen-activated protein kinase (MAPK) signaling cascade, which responds to extracellular signals and accumulates at the ‘fusion focus’ formed by actin aggregation, thereby initiating the fusion process [60,61]. At the structural level, the actin cytoskeleton and its adaptor proteins (e.g., spectraplakin/VAB-10A) provide a platform for fusion, which can recruit and anchor fusins (e.g., EFF-1) to specific plasma membrane sites; a microenvironment composed of membrane lipid components (e.g., phosphatidylserine) is essential for this process [62,63]. After fusion is completed, proteins such as EFF-1 can be actively cleared through dynein- and Rab5-dependent endocytosis pathways to prevent excessive fusion. An imbalance in this process leads to abnormal cell fusion [64]. Studies have shown that through cell fusion, the mitochondria of donor cells (such as human mesenchymal stem cells) can be transferred to recipient cells (such as cardiomyocytes) and participate in their energy metabolism [59]. However, since fusion is accompanied by the hybrid exchange of various cytoplasmic components, the specific role and functional effects of mitochondrial transfer in this process have not been fully elucidated and are still the focus of future research. Studies have also reported that cell fusion cannot be classified as a type of mitochondrial transfer because no clear receptor or donor cell is involved [20,65].

#### 2.2.5. Free Mitochondria Release and Capture

The release and capture of free mitochondria is a process in which cells actively expel mitochondria or their components (such as mtDNA) directly into the extracellular space under stress conditions (such as severe oxidative damage, specific death signals or pathogen stimulation). This process can be achieved by fusion with the plasma membrane after cytoplasmic vacuolation, the autophagic lysosome pathway or direct extrusion without membrane carriers. These released free mitochondria can then be captured by surrounding cells [66,67]. The mechanism of mitochondrial capture is not clear. Studies have shown that heparan sulfate (HS) may play a key role in this process: as a specific adhesion factor on the cell surface, it recognizes and binds to intact mitochondria without affecting the phagocytosis of other foreign bodies. The physiological and pathological significance of the release and capture of free mitochondria is dual: on the one hand, it may serve as a metabolic rescue mechanism to provide functional mitochondria for recipient cells in the tissue damage area for repair; on the other hand, the release of dysfunctional mitochondria can also activate harmful reactions such as neuroinflammation, which plays a key role in many pathological processes, such as neurodegenerative diseases, infection response and extreme stress defense of bone tissue [66,68,69].

### 2.3. Core Regulatory Molecules of Mitochondrial Transport

Intercellular mitochondrial transport is not a spontaneous process but is precisely driven by a set of highly conserved core molecular mechanisms in which the MIRO1/2-TRAK1/2 adaptor complex holds a central regulatory position. MIRO is a major regulator of mitochondrial dynamics and is responsible for the coordination of mitochondrial transport, homeostasis, degradation, morphogenesis, genetics, and contact with the endoplasmic reticulum [70,71,72]. From the N-terminus to the C-terminus, MIRO contains a first GTPase domain (nGTPase), two pairs of EF-hand structures, a second GTPase domain (cGTPase), and a transmembrane helix inserted into the mitochondrial outer membrane. The EF hand of MIRO has a unique structure, which is called the ELM domain (ELM1 and ELM2). Each domain contains a typical Ca^2+^-binding EF hand, a ‘hidden’ EF hand that does not bind to Ca^2+^, and a ligand mimic helix that occupies a hydrophobic pocket [73,74]. The regulatory effect of MIRO on mitochondrial dynamics depends on its nucleotide and Ca^2+^ binding state [70,75]. MIRO includes two paralogous proteins, MIRO1 and MIRO2, which can recruit several proteins to mitochondria [76]. Among them, myosin-19 mediates actin-based mitochondrial dynamics, while transport kinesin-binding protein (TRAK) recruits the microtubule-based motor protein kinesin-1 and dynein-kinesin activator protein complexes for long-distance transport [77,78]. TRAK also has two paralogous proteins, TRAK1 and TRAK2. The N-terminus of TRAK has a conserved coiled-coil domain, which is used to bind and activate kinesin-1 and the dynein-kinetin activator protein complex. In addition, the C-terminus is less conserved and mainly unstructured [79]. TRAK1 forms a complex with MIRO1 and is cotransported along microtubules driven by kinesin-1 or dynein-kinesin activator protein [80]. MIRO1 can bind to two amino acid sites on TRAK1. The initial recruitment of TRAK to mitochondria may be mediated by site-1, but TRAK in this state is in a self-inhibitory conformation; its complete activation and further recruitment of microtubule motors depend on site-2-mediated interactions [81].

Studies have confirmed that Cx43 is a key molecule that regulates the formation and function of TNTs. In an airway allergic inflammation model, the formation of TNTs between induced pluripotent stem cell-derived mesenchymal stem cells and airway epithelial cells depends on the Cx43 gap junction channel; downregulation of Cx43 expression not only significantly inhibits mitochondrial transmission between cells but also blocks the protective effect of mesenchymal stem cells on lung tissue [82]. In breast cancer cells, the functions of Cx43 and its related signaling pathways (such as Rho-associated coiled-coil kinase, protein kinase A, focal adhesion kinase and p38) in regulating the formation of TNTs have been shown to be nonclassical [83]. Knockout of the CX43 gene in human trabecular meshwork cells also results in a significant decrease in the number of TNTs. This evidence suggests that the Cx43 gap junction channel is likely involved in the formation of TNTs by mediating intercellular ion exchange (especially Ca^2+^) to coordinate cell–cell interactions; studies have shown that calcium signal exchange occurs between cells connected by TNTs [25]. In addition, a functional gap junction structure has also been observed at the end of the membrane extension of the TNTs, further supporting this hypothesis [84]. Moreover, the Cx43 gap junction channel is involved in the exchange of reactive oxygen species between cells and may even directly mediate the transfer of mitochondria through the channel. This may also be one of the potential mechanisms underlying the formation of TNTs and their ability to mediate mitochondrial intercellular transport [85,86].

Heparan sulfate (HS) is a sulfated glycosaminoglycan. It is usually covalently bound to the core protein to form heparan sulfate proteoglycans, which are distributed on the cell surface and in the extracellular matrix. Heparan sulfate plays important roles in regulating cell communication, embryonic development, homeostasis and many diseases [87]. HS plays an important role in the capture of free bodies and mitochondrial vesicles by receptor cells [68]. The mechanism may be related to the direct regulation of endosomal membrane budding and exosome formation by transmembrane heparan sulfate proteoglycans (such as multiligand glycans) connected to the ESCRT mechanism component ALIX through its intracellular adaptor protein multiligand protein [88]. Studies have shown that the sulfation modification of HS at its 6-O position is crucial for the capture of free mitochondria. Gene knockout, inflammatory stimulation, obesity modeling and heparin intervention experiments have confirmed that the level of HS on the surface of macrophages is directly related to the level of mitochondrial uptake [68]. The uptake of β-cell-derived MEVs by macrophages is significantly reduced by the use of HS-degrading endoglycosidases [89]. These findings suggest that HS mediates mitochondrial transfer between cells and suggests clinical heparin therapy may affect this process.

Mitochondrial transfer is closely related to the regulation of mitochondrial fission by Drp1. Mitochondrial fission is a complex process involving multiple steps upstream and downstream of Drp1 recruitment. It begins with the correct assembly of Drp1 on the mitochondrial membrane and is gradually promoted by Drp1-driven membrane contraction, eventually leading to membrane rupture [90]. Drp1 is a key power-related GTPase that performs mitochondrial fission. Its structure contains an N-terminal GTPase domain, intermediate domain, variable domain (VD) and C-terminal GTPase effector domain (GED) [91]. It lacks a domain that directly interacts with membrane lipids; thus, it must rely on a receptor protein on the mitochondrial outer membrane (OMM) to be recruited to the mitochondria and assembled into a functional fission ring [92]. To date, four major OMM adaptor proteins have been identified, namely, Fis1, Mff, Mid49 and Mid51, which interact with Drp1 in different ways to regulate its recruitment, oligomerization and activation. Fis1, which is anchored to the OMM through its C-terminal transmembrane domain and interacts with Drp1 through the N-terminal repetitive motif as a scaffold, was the first discovered Drp1 recruitment factor [93]. Studies suggest that Fis1 may not be necessary for the recruitment of Drp1. It may play a regulatory role in the abnormal interaction between Drp1 and Fis1 in specific physiological processes or specific pathological conditions rather than being the main constitutive recruitment receptor [94]. Mff is considered to be the main receptor for the recruitment of Drp1 to mitochondria in mammals. Its structural features include C-terminal transmembrane anchoring and N-terminal cytoplasmic-facing short repeat motifs and coiled-coil domains [95]. Functionally, overexpression of Mff strongly recruits cytoplasmic Drp1 to mitochondria and triggers significant division, whereas its deletion severely impairs the mitochondrial localization of Drp1 and inhibits division [96]. Mid49 and Mid51 are a pair of homologous proteins that exist only in vertebrates. They are anchored to the OMM through the N-terminal transmembrane domain and can recruit cytoplasmic Drp1 in a manner independent of Fis1 and Mff. However, their regulation of Drp1 activity and division shows dual and dynamic characteristics. In the steady state, overexpression of Mid49 or Mid51 usually seals Drp1 on the surface of mitochondria but inhibits its GTPase activity, leading to mitochondrial fusion or elongation and inhibiting division [97,98]. Drp1-mediated mitochondrial fission occurs when actin filaments first promote the preassembly and oligomerization of cytoplasmic Drp1, after which oligomerized adaptor proteins (such as MFF) efficiently recognize and recruit these Drp1 precursor complexes. After being recruited, the adaptor protein further stimulates the GTPase activity of Drp1 and drives it to assemble into a higher-order helical structure on the OMM. Finally, by hydrolyzing GTP to achieve physical contraction and division of the mitochondrial membrane, Drp1 induces conformational changes and mechanical forces [99]. Studies have shown that enhanced Drp1-Mff signaling enhances the ability of macrophages to transfer healthy mitochondria to endothelial and neuronal cells, thereby promoting bone healing [100].

## 3. Cell-Specific Mechanism Through Which Mitochondrial Transport Regulates Bone Metabolism Homeostasis

**Figure 2:**
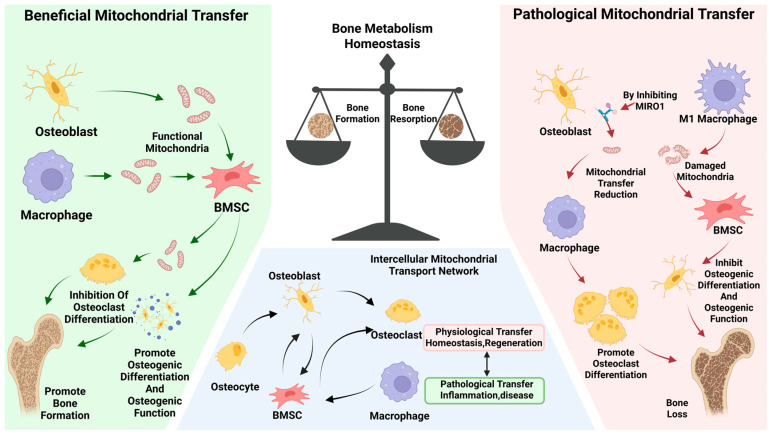
Intercellular mitochondrial transfer network in the bone microenvironment. Intercellular mitochondrial transfer plays a crucial regulatory role in bone metabolic homeostasis. It constitutes a complex mitochondrial transport network among various bone-related cells, including osteoblasts, osteoclasts, bone marrow mesenchymal stem cells (BMSCs), and macrophages. This transfer occurs under both physiological and pathological conditions. Beneficial mitochondrial transfer can promote bone formation and maintain or restore bone homeostasis by modulating the functions of BMSCs, osteoclasts and their precursor cells. Conversely, pathological mitochondrial transfer can disrupt bone homeostasis and lead to bone loss through the transfer of dysfunctional mitochondria into BMSCs or through the inhibition of normal mitochondrial transfer processes. Created in BioRender. Wang, X. (2026) https://BioRender.com/z7yu572.

The skeletal system is composed of a variety of bone cells and hematopoietic cells, and the dynamic interaction between them is crucial for maintaining the homeostasis of bone metabolism. Mesenchymal stem cell (MSC)-derived bone cells and hematopoietic stem cell (HSC)-derived bone marrow cells are the two core functional cell lineages [101]. Bone lineage cells, including bone precursor cells, osteoblasts and osteocytes, mainly mediate bone formation. Moreover, increasing evidence has shown that these cells are important regulatory units of the bone marrow microenvironment and can participate in the construction of bone marrow hematopoietic niches and affect the differentiation and lineage distribution of myeloid and lymphoid cells [102]. In addition, bone marrow cells can further differentiate into osteoclast lineage cells to mediate bone resorption and interact with osteoblast lineage cells in time and space during bone remodeling [103]. The abnormal energy metabolism of the above cell lineages can disrupt bone homeostasis, which in turn can induce and promote the development of bone diseases [104]. Although the complex signal communication between these cells has been reported, the underlying regulatory mechanism has not been fully elucidated. As mitochondria are dynamic organelles with both energy conversion and signal transduction functions, their transfer between cells has gradually become regarded as an important method of information transmission. It can affect gene transcription and translation by comprehensively regulating the cascade activation of multiple signaling pathways in recipient cells and ultimately determining the cell phenotype [105,106]. However, the existing reviews have not comprehensively summarized this regulatory process. Therefore, the following section focuses on the core regulatory network through which mitochondrial transport regulates osteoblast function, osteoclast function and bone metabolism homeostasis and reviews its molecular mechanism (Figure 2).

### 3.1. Mitochondrial Transport Regulates Osteoblast Differentiation, Mineralization and Functional Maintenance

Osteoblast differentiation and bone matrix mineralization are highly dependent on cellular energy metabolism. Mitochondria, as the core organelles involved in energy metabolism and signal integration, play key roles in the regulation of osteogenic differentiation [107,108]. Studies have shown that the mitochondrial transport process between myeloid cells and osteogenic lineage cells in bone marrow depends on the expression of the MIRO1 protein. Moreover, macrophages are important mitochondrial donor cells. After mechanical stimulation activates Drp1 and mediates mitochondrial fission, macrophages can transfer mitochondria to BMSCs by releasing mitochondria-rich extracellular vesicles or direct cell-to-cell contact to increase their osteogenic potential and promote bone formation [23,52]. Inflammatory macrophages can transmit damaged mitochondria to BMSCs in the form of MEVs, thereby destroying the mitochondrial dynamics of BMSCs, inhibiting the formation of donut-shaped mitochondria, and ultimately damaging their bone formation ability. Further studies have revealed that macrophage-derived MEVs can significantly increase the expression of lipid carrier protein 2 (LCN2) in BMSCs. LCN2 interferes with mitochondrial morphological remodeling in BMSCs by promoting the degradation of OMA1 protein and causing the accumulation of OPA1 protein, resulting in decreased osteogenic function. It is worth noting that inhibition of LCN2 can effectively reverse osteogenic dysfunction in BMSCs and reduce alveolar bone loss in periodontitis models [54]. Among them, M2 macrophage-derived mitochondria can maintain cell metabolic homeostasis by activating signaling pathways such as p38-MAPK and further promote bone tissue regeneration; mitochondrial transfer itself can also significantly increase the level of oxidative phosphorylation (OXPHOS) and ATP production in BMSCs, increase cell proliferation, migration and osteogenic differentiation, and ultimately promote bone defect repair [22,109]. In addition, the introduction of osteoblast mitochondria into MSCs promotes bone formation through the bone morphogenetic protein 2 (BMP2)-Wnt/β-catenin-calcium input axis [110]. On the basis of the above mechanism, researchers have constructed a new hydrogel loaded with MIRO1 protein and functionalized it with ferric oxide nanoparticles (KGM-PEG-SPIONs). The former can enhance osteogenic differentiation and accelerate the regeneration of skull defects in mice by promoting the mitochondrial transfer of macrophages to BMSCs. The latter can transfer mitochondria to senescent BMSCs through the CX43 gap junction to restore its mitochondrial membrane potential, ATP production, calcium homeostasis and osteogenic differentiation ability [111,112].

### 3.2. Mitochondrial Transport Regulates Osteoclast Activation and Bone Resorption Activity

Osteoclasts are multinucleated cells derived from the mononuclear-macrophage system, and their differentiation, maturation and bone resorption function are highly dependent on mitochondrial metabolism. The process of bone resorption requires a large amount of ATP to maintain proton pump activity and cytoskeletal remodeling. Therefore, the functional status of mitochondria directly determines the bone resorption capacity of osteoclasts [113,114]. In recent years, studies have confirmed that the mitochondrial transport of osteoblast lineage cells to myeloid cells plays a key role in maintaining bone metabolism homeostasis; when the mitochondrial transport-related protein MIRO1 in osteoblasts is absent, the transfer of mitochondria to myeloid cells is significantly reduced, which in turn promotes the differentiation of myeloid cells into the osteoclast lineage and enhances bone resorption activity. Mechanistic studies have shown that the above process can maintain the survival and differentiation potential of osteoclast precursor cells by regulating glutathione metabolism and inhibiting ferroptosis signals [23]. Mesenchymal cell-derived mitochondria can increase oxidative stress in mononuclear macrophages and restore mitochondrial function by upregulating SOD expression, thereby regulating osteoclastogenesis and inflammatory bone resorption [115]. Exosomes secreted by myoblasts can inhibit the differentiation of mononuclear macrophages into osteoclasts by inhibiting mitochondrial biosynthesis [116]. On the basis of the above mechanism, researchers have constructed artificial cell microspheres (Fmito@ACs) carrying the mitochondria of fetal rat mesenchymal stem cells. This system can improve the aging phenotype of older bone marrow mesenchymal stem cells and inhibit osteoclast formation by promoting mitochondrial fusion and aerobic glycolysis [117].

### 3.3. Mitochondrial Trafficking Between Osteoblasts, Osteoblasts, and Osteoclasts: The Core Regulatory Network of Bone Metabolism Homeostasis

The synergistic effect of multiple cells in the bone marrow is the key to maintaining bone metabolism homeostasis. Bone homeostasis depends mainly on the dynamic balance between osteoclast bone resorption and osteoblast bone formation and is closely related to cell energy metabolism. Studies have confirmed that intercellular mitochondrial transfer can occur widely in vivo and in vitro. However, previous studies have focused on obtaining mitochondria from donor cells such as mesenchymal stem cells (MSCs) from damaged cells or tumor cells to meet their own metabolic and proliferation needs. However, few studies have investigated the transfer of mitochondria to MSCs and have suggested that mitochondria are involved in the antiapoptotic and prodifferentiation effects of stem cells [118,119,120]. Levoux et al. reported that platelets can enhance the wound repair ability of MSCs through mitochondrial transfer and that platelets with abnormal mitochondrial function significantly weaken their therapeutic effects, suggesting that the type and functional status of donor cells can determine the biological effects of recipient cells after mitochondrial transfer [69]. Cai et al. further confirmed that the infusion of abnormal mitochondria derived from M1 macrophages can induce an osteoporosis-like phenotype in mice, whereas normal mitochondrial transplantation can effectively alleviate bone loss after ovariectomy (OVX), indicating that mitochondrial sources directly affect bone metabolism homeostasis. Mechanistically, the abnormal transfer of M1 macrophages to the mitochondria of MSCs can lead to an increase in reactive oxygen species (ROS) in recipient cells, a decrease in mitochondrial membrane potential (MMP), a decrease in the oxygen consumption rate, and an increase in the degree of glycolysis, resulting in metabolic reprogramming and osteogenic differentiation disorders in MSCs. Studies have also shown that abnormal mitochondrial transfer can significantly upregulate the accumulation of succinic acid, an intermediate product of the tricarboxylic acid cycle, in MSCs. Succinic acid can stabilize Hif-1α by inhibiting the prolyl hydroxylase domain (PHD), thereby promoting the transcription of inflammatory genes such as IL-1β, which aggravates metabolic disorders and inflammatory responses [121]. It is worth noting that normal mitochondrial transplantation does not increase succinate levels under physiological conditions, whereas MSC-derived exosomes can promote angiogenesis and fracture repair by activating the Hif-1α/Vegfa pathway, which is consistent with the bone recovery effect of OVX [122]. The above evidence shows that osteogenesis, osteoclasts and mitochondrial cross-transport between bone cells constitute the core regulatory network of bone metabolism homeostasis through the regulation of the metabolic status of MSCs, inflammatory signals and osteogenic differentiation, and abnormal bone transport is an important mechanism for the induction of bone metabolic diseases.

## 4. Abnormal Mitochondrial Transport Mediates the Pathological Mechanism of Bone Metabolic Diseases

**Figure 3:**
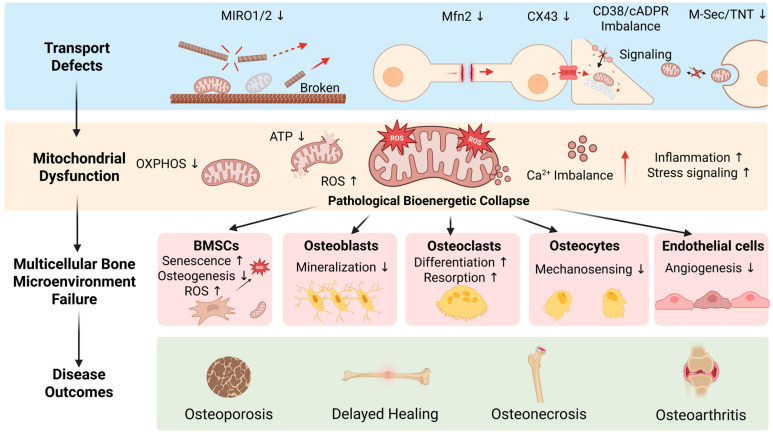
Diagram of the pathological mechanism of bone diseases driven by abnormal mitochondrial transport. Structural or functional impairment of the mitochondrial transfer network is a critical component of the pathogenesis of metabolic bone diseases. Abnormalities in mitochondrial transport—including the blockade of TNT formation or the downregulation or mutation of key regulatory proteins such as MIRO1 and Mfn2—result in mitochondrial dysfunction within recipient cells. Consequently, damaged bone marrow mesenchymal stem cells (BMSCs), osteoblasts, or osteocytes are not replenished by exogenous healthy mitochondria. This deprivation leads to insufficient ATP production and excessive accumulation of reactive oxygen species (ROS), subsequently triggering apoptosis or premature cellular senescence. Furthermore, impaired mitochondrial transfer from osteolineage cells to myeloid cells relieves the inhibitory constraints on osteoclast differentiation, leading to osteoclast overactivation. Ultimately, the disruption of this intercellular metabolic communication disrupts the balance between bone formation and bone resorption, directly driving pathological processes such as osteoporosis, glucocorticoid-induced bone loss, and cartilage degeneration in osteoarthritis. Created in BioRender. Wang, X. (2026) https://BioRender.com/thjwxno.

Mitochondria are the core of cell energy supply and signal regulation. Directional transport, intracellular distribution and intercellular transmission of mitochondria in bone tissue are key for maintaining the dynamic balance between osteogenesis and osteoclasts and ensuring the homeostasis of bone remodeling. The abnormal expression of key proteins or related transport pathways that regulate mitochondrial transport can lead to mitochondrial distribution disorder, dysfunction and intercellular transmission disorder in bone cells, which in turn can lead to energy metabolism disorder, increased oxidative stress levels, calcium signal abnormalities and differentiation dysfunction in bone cells. These pathological changes inhibit the bone formation of osteoblasts, abnormally increase the bone resorption activity of osteoclasts, and disrupt the metabolism and immune homeostasis of the bone marrow microenvironment, ultimately disrupting the balance of bone remodeling and inducing osteoporosis, osteoarthritis, bone dysplasia and other bone metabolic diseases. It is worth noting that on the basis of the pathological mechanism of mitochondrial transport disorders, a series of innovative therapeutic strategies are being developed, such as the use of engineered macrophages for targeted mitochondrial delivery, the construction of smart hydrogel scaffolds to regulate mitochondrial transfer, and the design of artificial cell carriers to achieve bone-targeted mitochondrial transplantation, which provide a new perspective for the treatment of refractory bone diseases [111,117,123]. The specific pathological mechanism of abnormal mitochondrial transport-mediated development of bone metabolic diseases is comprehensively described below (Figure 3).

### 4.1. Osteoporosis (OP)

OP is a metabolic bone disease that is mediated by multiple mechanisms. Its pathological characteristics include normal calcification of bone tissue and a constant ratio of calcium salt to matrix but a significant reduction in total bone tissue per unit volume. OP is closely related to the abnormal enhancement of bone resorption. In recent years, studies have shown that OP is no longer a disease exclusive to the elderly population. Its pathogenesis can involve nutritional imbalance (such as protein, vitamin C or calcium deficiency), weakening of bone mechanical stimulation caused by muscle atrophy, imbalance of bone formation and bone resorption, and other factors. The etiology of special types of OP, such as adolescent OP, remains to be further elucidated. Therefore, in-depth analyses of the pathogenesis of OP and a reduction in its incidence have become important issues in the field of bone diseases.

Mitochondrial transmission plays a key role in maintaining bone homeostasis. Osteoblastic lineage cells embedded in mineralized bone matrix can achieve mitochondrial intercellular transmission through the dendritic cell network and then regulate the balance of bone remodeling through metabolic interactions. In the pathological state of osteoporosis, disorders of mitochondrial transmission between cells, destruction of TNTs structure and a decrease in MIRO-TRAK axis function lead to disorders of bone tissue metabolic homeostasis and promote the occurrence and development of OP. Under normal circumstances, macrophages in the bone marrow microenvironment can promote osteogenic differentiation by transmitting mitochondria to MSCs. In osteoporosis, the damage to mitochondria transmitted by M1 macrophages to MSCs can lead to MSC damage and metabolic remodeling through the accumulation of succinic acid, affecting the TCA cycle and regulating the expression of Hif-1α [121]. These findings indicate that increased abnormal mitochondrial transfer changes the metabolic state of MSCs, which in turn leads to disordered bone formation and differentiation. Osteogenic lineage cells (including bone progenitor cells and osteoblasts) rely on the MIRO1 protein to unidirectionally transfer functional mitochondria to myeloid cells in the bone marrow, especially monocytes/macrophages; when this metastasis is impaired, such as when MIRO1 is knocked out, differentiation of myeloid cells into osteoclast lineages is promoted, osteoclast activity is enhanced, and bone resorption and osteoporosis are increased. After the transferred mitochondria are received by osteoclast precursor cells, they inhibit glutathione synthesis and induce ferroptosis, thereby inhibiting the survival and function of osteoclasts. In glucocorticoid-induced osteoporosis (GIOP), glucocorticoid therapy inhibits the expression of MIRO1 in osteoblasts, impairs mitochondrial metastasis, and exacerbates glutathione depletion, whereas drug consumption of glutathione can alleviate the progression of osteoporosis. Therefore, the mitochondrial transfer of bone lineage cells to bone marrow cells inhibits bone resorption by regulating glutathione metabolism and ferroptosis. As a key mediator, MIRO1 dysfunction directly leads to mitochondrial transmission disorder and is involved in the pathological process of osteoporosis [23].

### 4.2. Osteonecrosis of the Femoral Head (ONFH)

ONFH is a refractory disease in the field of orthopedics. The pathogenesis of ONFH is not completely clear. Its core feature is the abnormal microenvironment of the femoral head, which eventually leads to the death of bone cells and the collapse of bone tissue. In recent years, studies have confirmed that mitochondrial damage is closely related to osteocyte senescence and apoptosis. The synergistic effect of hypoxia and glucocorticoids (GCs) is the core mechanism through which mitochondrial function is inhibited and osteocyte apoptosis is induced. In vivo experiments have shown that improving mitochondrial transport function can effectively restore the mitochondrial function of bone cells, opening up a new direction for the study of the pathogenesis of ONFH [124].

Disorders of mitochondrial damage, dysfunction and mitochondrial transport in osteocytes, including ATP synthesis disorders, calcium metabolism imbalance, abnormal inflammatory responses, excessive ROS release and mitochondrial intracellular distribution imbalance, are important causes of osteocyte apoptosis during ONFH. Bone-related cells, immune cells, and endothelial cells in bone tissue share the same microenvironment and participate in the regulation of bone homeostasis. Osteocytes rely on normal mitochondrial function and directional transport to maintain homeostasis. Studies have shown that stressed osteocytes release adenosine diphosphate (ADP), which triggers the mitochondrial transfer of healthy osteocytes to stressed osteocytes by activating P2Y2 and P2Y6 receptor signaling to restore their oxygen consumption rate and alleviate ROS accumulation. This finding reveals the signaling mechanism that initiates mitochondrial rescue transfer under stress conditions such as hypoxia [124]. Moreover, glucocorticoids can destroy the connection between mitochondria and the cytoskeleton by degrading mitochondrial transport-related regulatory proteins such as MFN2 and hindering mitochondrial intracellular transport and intercellular transport [11]. Hypoxia and glucocorticoid-mediated mitochondrial dysfunction directly lead to insufficient energy supply and ROS accumulation in osteocytes, eventually inducing osteocyte apoptosis and promoting the progression of ONFH.

### 4.3. Osteoarthritis (OA)

OA is a heterogeneous syndrome that is closely related to oxidative stress and synovial inflammation. Mitochondrial dysfunction is among the core mechanisms that mediate OA pathological progression. ROSs are widely considered to contribute to chondrocyte damage and osteoarthritis progression. Excessive production of ROSs destroys joint homeostasis, triggers apoptosis and accelerates metabolic degradation, thereby damaging various structures of the joint. In recent years, studies have shown that mitochondrial dysfunction is common in OA chondrocytes and is positively correlated with chondrocyte hypertrophy and apoptosis. This mechanism has been verified in a variety of OA animal models and clinical samples. Mitochondrial homeostasis is the basis of the normal function of chondrocytes, and abnormalities in mitochondrial homeostasis can lead to mitochondrial dysfunction, which in turn induces chondrocyte injury and apoptosis and promotes the development of OA.

Given the critical role of mitochondrial homeostasis in chondrocyte function, restoring mitochondrial dysfunction through intercellular mitochondrial transport has become a rapidly evolving research hotspot in the field of osteoarthritis therapy [125,126]. In the inflammatory environment, the process of mitochondrial transport from MSCs to chondrocytes is significantly increased, and Cx43 plays a key role in this process [127]. After they contact damaged cartilage, MSCs are located at the damaged site and transmit mitochondria to chondrocytes through cell protrusions [125]. Studies have shown that MSCs can actively transport their own mitochondria to the chondrocytes of OA patients, thereby improving the oxidative phosphorylation (OXPHOS) ability of recipient cells and promoting metabolism to switch from compensatory glycolysis to a more efficient oxidative metabolic state, directly correcting the defect of insufficient energy in recipient chondrocytes. In addition, mitochondrial transport promotes mitochondrial fusion in receptor cells and inhibits OA-related pathological division and mitophagy, thereby protecting cells from apoptosis. Moreover, the receptor chondrocytes highly express superoxide dismutase (SOD2) mRNA and protein, thereby enhancing their antioxidant defense ability, effectively reducing the oxidative damage mediated by ROSs produced by mitochondria, and ultimately reducing the apoptosis of chondrocytes under stress conditions [126]. Mitochondrial transport can also promote cartilage regeneration by affecting the joint microenvironment. Mei et al. found that mesenchymal stem cells can transport functional mitochondria to dendritic cells (DCs) through the mitochondrial vesicle pathway. Functional mitochondria can ameliorate the decreased mitochondrial membrane potential of DCs caused by LPS stimulation and reduce the expression of surface activation markers and inflammatory cytokines by enhancing DC autophagy. The treatment of chondrocytes with the culture supernatant of EV-treated DCs can promote the recovery of chondrocytes and ultimately alleviate inflammation and injury to the temporomandibular joint [128]. However, mitochondrial transport may also be a proinflammatory factor under certain conditions. Studies have shown that a high cholesterol environment stimulates osteocytes in subchondral bone tissue to transport specific subsets of mitochondria to chondrocytes through the TNT pathway, thereby activating the cGAS-STING pathway in chondrocytes and causing inflammation and cartilage destruction [129].

### 4.4. Diabetic Bone Disease

Diabetic bone disease is a complication of the skeletal system caused by diabetes. It is characterized by normal or increased bone mineral density, but the microstructure of the bone is destroyed, and the bone quality is significantly degraded, resulting in a significantly greater risk of fracture in patients with type 2 diabetes than in nondiabetic people. Mitochondrial transfer can have a dual effect on cell metabolism, especially when macrophages release dysfunctional mitochondria, which can induce proinflammatory responses in endothelial cells or lead to inflammatory responses in neurons [130,131]. As one of the important characteristics of diabetes, elevated levels of systemic circulation mitochondria may be involved in and mediate the pathophysiological process of diabetic bone disease [132]. Recent studies have shown that silicon can enhance the ability of macrophages to transfer healthy mitochondria to endothelial and neuronal cells through the Drp1-Mff signaling pathway, thereby promoting bone healing [100]. As the main receptor of Drp1 in most mammalian cells, the Mff pathway plays a key role in regulating mitochondrial size, maintaining mitochondrial integrity and promoting mitochondrial transport [133]. Promoting the exchange of healthy mitochondria can not only increase the damage to related cells caused by the diabetic microenvironment but also provide a favorable microenvironment for bone healing. Therefore, enhancing healthy mitochondrial transfer may improve diabetic bone disease by optimizing intercellular communication.

## 5. Mitochondrial Transport-Targeted Intervention Strategies and Translational Prospects in Bone Metabolic Diseases

**Figure 4:**
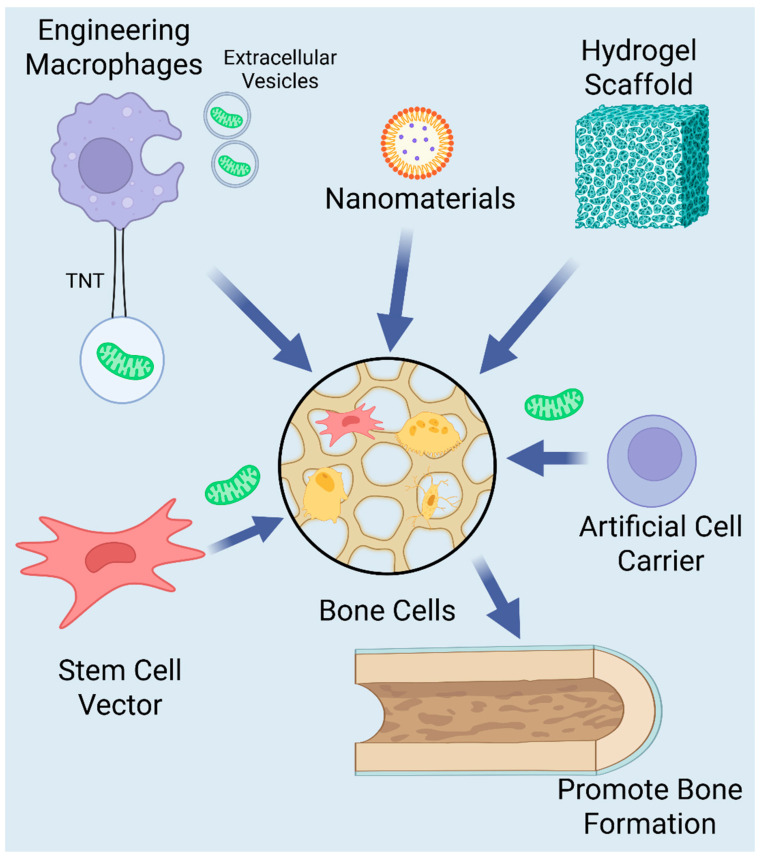
Therapeutic strategies targeting mitochondrial transfer for bone regeneration and metabolic bone diseases. Artificial mitochondria transfer. Mitochondria are isolated from healthy donors (e.g., BMSCs) in vitro and directly delivered to damaged cells or bone defect regions using specific cell types or artificial microspheres as carriers. This approach rapidly restores local aerobic metabolism and cellular viability. Intervention in transfer channels and molecular mechanism: Drugs are delivered via hydrogels, nanomaterials, or other delivery systems to increase the efficiency of endogenous intercellular mitochondrial transfer. Optimization of stem cell therapy: Mitochondrial preconditioning or genetic modification of BMSCs prior to transplantation improves their survival in the harsh posttransplantation microenvironment and enhances their ability to transfer mitochondria to host cells, thereby significantly accelerating in situ bone defect healing and bone regeneration. Created in BioRender. Wang, X. (2026) https://BioRender.com/gyf3cs6.

Mitochondrial dysfunction is an important pathological link in many bone metabolic diseases, such as osteoporosis, osteoarthritis, and osteonecrosis of the femoral head. Intervention strategies targeting mitochondrial transport have become a research hotspot for the precise treatment of bone metabolic diseases. The core idea is to restore mitochondrial homeostasis and reverse bone metabolic imbalance by transmitting functional mitochondria and regulating transport-related molecules and pathways. Moreover, these interventions have good clinical translational potential. At present, the intervention strategies can be divided into three main categories: mitochondrial transplantation, stem cell-mediated mitochondrial delivery repair and precise nanocarrier-mediated intervention. The following section will comprehensively discuss the targeted mitochondrial transport intervention strategies for the treatment of bone metabolic diseases (Figure 4).

### 5.1. Mitochondrial Transplantation

Mitochondrial transplantation is a new treatment strategy for bone diseases. Its core mechanism relies on the regulation of extracellular vesicle transport and intercellular mitochondrial transfer, which provides important support for bone homeostasis maintenance and bone regeneration. The accumulation of many mitochondrial components in the bone matrix reflects its active participation in osteoblast maturation and regulation of mineral homeostasis. Fully differentiated osteoblasts can actively export intact mitochondria and mitochondrial-derived vesicles (MDVs) to enhance the bone formation process of mesenchymal stem cell precursors [10]. Mitochondrial morphological plasticity is closely related to bone formation and differentiation. Differentiation signals trigger mitochondrial network reorganization. Mitochondrial output capacity can be significantly enhanced by regulating the fusion-fission balance, which provides a molecular basis for mitochondrial transplantation and is expected to stimulate bone formation and prevent osteoporosis-related bone degradation.

Osteoblasts are terminally differentiated cells; their channel network system provides an important carrier for mitochondrial transfer and is a model system for mitochondrial transplantation therapy research. Under metabolic stress, bone cells can restore energy homeostasis by internalizing the mitochondria of surrounding functional bone cells. This process depends on the endoplasmic reticulum–mitochondrial membrane contact site and regulation mediated by Mfn2 and VAPB, in which Mfn2 is responsible for establishing endoplasmic reticulum–mitochondrial connections and promoting mitochondrial exchange between cells. Decreases in age-related Mfn2 expression can lead to mitochondrial transport defects and decreased transfer efficiency, which in turn leads to osteocyte apoptosis and metabolic disorders and promotes the development of osteoporosis, suggesting that Mfn2 can be used as a potential regulatory target for mitochondrial transplantation therapy [11]. As a core regulator of microtubule-associated mitochondrial transport, MIRO1 is involved in the mitochondrial transfer of the osteocyte–vascular axis and is essential for maintaining vascular integrity and bone repair. Bone cells can be transferred to endothelial cells through MIRO1-mediated mitochondria to maintain microvascular structure and function. The loss of bone cells leads to vascular degeneration. Endothelial cells that receive bone cell-derived mitochondria can restore function, increase angiogenesis and bone repair, and provide a new path for mitochondrial transplantation in the treatment of vascular-related bone diseases (such as osteoporosis and impaired fracture healing) [12].

The use of cells or artificial cells as carriers of mitochondrial transfer systems has become important. Nie et al. constructed an artificial cell microsphere (Fmito@ACs) system containing mesenchymal stem cell mitochondria. This system enhances mitochondrial fusion and aerobic glycolysis by targeting mitochondria to senescent BMSCs, thereby alleviating the senescence of recipient cells, promoting osteoblast differentiation and inhibiting osteoclast formation [117]. Cheng et al. used red blood cells as carriers to load mitochondria derived from myocardial cells and constructed a mitochondrial-loaded red blood cell (MiLE) system that can maintain mitochondrial function for a long time and achieve targeted delivery of inflammatory sites. By delivering mitochondria and oxygen to macrophages, MiLE reverses the metabolic reprogramming of lipopolysaccharides and inhibits osteoclast differentiation, thereby effectively alleviating inflammatory bone loss. Mechanistically, MILE can promote macrophage polarization from proinflammatory M1 macrophages to anti-inflammatory M2 macrophages by inhibiting macrophage glycolysis, promoting oxidative phosphorylation, and inhibiting osteoclast differentiation [134]. Wang et al. constructed a live mitochondrial delivery system composed of engineered CXCR4 macrophages loaded with nanozyme-functionalized mitochondria (CM-MTBM). CM-MTBM promotes the metabolic recovery and redox balance of BMSCs by delivering functional mitochondria to BMSCs, restoring mitochondrial respiration, promoting osteoblast differentiation, and alleviating oxidative apoptosis of BMSCs. Mechanistically, CM-MTBM activates mitochondrial oxidative metabolism, inhibits inflammation and aging-related signals, and achieves the coordination of metabolic and osteogenic activation [123]. New hydrogels, such as the Gel@MDI hydrogel and GAD/Ag-pIOPN hydrogel scaffold, developed through biomaterial engineering, promote the directional mitochondrial delivery of macrophages to BMSCs, alleviate inflammation-mediated metabolic inhibition, and enhance bone formation and differentiation of BMSCs. Animal experiments have confirmed that these hydrogels can promote fracture healing, providing preclinical support for the use of mitochondrial transplantation in the treatment of osteoporotic fractures. Meanwhile, both hydrogels demonstrate superior mitochondrial transfer efficiency, higher biocompatibility, and enhanced immunomodulation by targeting the transfer of mitochondria from macrophages to bone marrow mesenchymal stem cells, highlighting the broad application potential of these novel hydrogels in the treatment of bone metabolic diseases [111,135].

However, there are still many challenges hindering the clinical application of mitochondrial transfer. Mitochondrial transfer requires the rapid isolation and loading of mitochondria [136], but at present, mitochondrial respiratory function is significantly lost approximately 2 h after isolation. In addition, isolation protocols must optimize the efficacy of isolated mitochondria by minimizing loss of function/viability and structure [137,138]. Current approaches to mitochondrial transfer lack target-cell specificity, and biotechnology-transferred mitochondria must cross cellular and body barriers to reach the target cell [139]. The efficiency of biotechnological mitochondrial transfer remains a major challenge. Available data show that the vector system used for mitochondrial transfer does not alter mitochondrial structure or function, but this critical question has not been fully explored. In fact, methods to assess the effects of these systems on mitochondrial activity and on the activity of recipient cells are inadequate and require further investigation [140]. In addition, most of the existing studies focus on using in vivo and in vitro pathological models to verify the effectiveness of mitochondrial transfer to restore mitochondrial dysfunction, but there is a serious lack of detailed evaluation of its safety. When few safety studies exist, they are often not in-depth or detailed. The overall effect of these novel agents on different organs and tissues in living models must be appreciated [141]. At the same time, most studies have focused on short-term efficacy monitoring; however, the true efficacy and therapeutic potential of this type of biotechnological therapy will need to be confirmed through longer-term follow-up [141,142]. Such long-term studies could likewise help to clarify whether regular infusions are needed to maintain successful management of the disease.

The possible immune response after allogeneic mitochondrial transfer is also an important challenge in the field of mitochondrial transfer. The immune response to mitochondrial transfer is currently subject to academic disagreement. Barbieri et al. suggested that isolated mitochondria exhibited very low immunogenicity, whether in autologous, congenic, or xenogeneic models. Experimental evidence showed that single or repeated injections did not induce significant allorecognition or adaptive immune responses, nor did they produce significant systemic inflammatory storm or anti-mitochondrial antibodies. This was mainly attributed to the lack of major histocompatibility complex (MHC) and costimulatory molecules in mitochondria. It is difficult to initiate the classical T-cell-mediated rejection pathway [142]. In contrast, Brennan et al. showed that a single injection of mitochondrial transfer induced an immune response. The team observed significant early rejection of cardiac allografts and proposed a mechanistic hypothesis: Extracellular mitochondria activate vascular endothelial cells and up-regulate the expression of inflammatory cytokines and chemokines. In turn, these activated endothelial cells accelerate the process of rejection by enhancing T-cell adhesion and infiltration into the graft tissue [143,144]. Therefore, the safety and efficacy of allogeneic mitochondrial transfer still need to be carefully evaluated from the perspective of immune response.

### 5.2. Stem Cell-Mediated Mitochondrial Delivery Repair

Mitochondria are the core organelles involved in cellular energy metabolism, and their functional damage can cause a variety of bone diseases (such as osteoarthritis and osteoporosis). Stem cell-mediated mitochondrial delivery repair has become important for the treatment of bone diseases. With respect to this strategy, mitochondrial transplantation is among the most promising clinical techniques. However, the wide application of mitochondrial transplantation is constrained by the limited supply of healthy mitochondria, and the dose of each injection/patient in clinical treatment needs to be as high as 10^9^. Therefore, the development of sustainable and easy-to-operate high-quality human mitochondrial generation methods has become a key barrier to promoting the transformation of stem cell-mediated mitochondrial delivery repair technology [145].

In response to this problem, Chen et al.’s team focused on the most commonly used stem cell types in MSC-stem cell-mediated mitochondrial delivery repair and proposed an efficient mitochondrial generation strategy involving the manipulation of mitochondrial biogenesis and regulation of the balance of organelles in MSCs. The researchers used a previously established formula to optimize a special medium (mitochondrial condition) and achieved an 854-fold increase in mitochondrial production compared with normal MSC culture within 15 days. These MSC-generated mitochondria not only significantly increased in number but also presented superior functional properties. The level of ATP production in these mitochondria was 5.71 times greater than that of normal mitochondria, indicating that these cells provide a high-quality and highly active mitochondrial source for stem cell-mediated mitochondrial delivery, which can effectively solve the problem of insufficient mitochondrial supply in clinical applications. At the mechanism level, this study revealed that AMPK pathway activation is the core regulatory mechanism through which MSCs efficiently generate mitochondria. The study also confirmed that MSCs can establish a new cell state under these culture conditions, characterized by enhanced proliferation and mitochondrial biogenesis, while other energy-consuming activities are inhibited. Together, MSCs provide an ideal cell microenvironment for the efficient synthesis of mitochondria. In addition, the therapeutic effect of these MSC-derived mitochondria in vivo was verified in a mouse osteoarthritis model. Through stem cell-mediated mitochondrial delivery, significant cartilage regeneration can be achieved within 12 weeks, further confirming the feasibility and value of this mitochondrial generation strategy in stem cell-mediated repair of bone metabolic diseases [146].

In general, this study proposes a new strategy for the construction of high-quality human mitochondria and clarifies the core role of MSCs in the efficient generation of functional intact mitochondria. It not only provides sufficient high-quality mitochondrial sources for stem cell-mediated mitochondrial delivery and repair but also reveals the molecular mechanism of organelle synthesis, laying an important foundation for promoting the transformation and application of stem cell-mediated mitochondrial delivery technology in the clinical treatment of bone metabolic diseases. However, clear evidence to confirm the efficient transfer efficiency and long-term retention ability of mitochondria in target cells is lacking. Whether mitochondria can continue to function in target cells after transplantation is unclear, and the relationship between their transfer efficiency and therapeutic effect is unknown, which has become a key limitation restricting the clinical transformation of this strategy. Therefore, future research should focus on developing an efficient and accurate advanced mitochondrial delivery system and comprehensively verifying the survival status, functional integrity and long-term effect of mitochondria in target cells after transplantation. These studies will further improve the technology for mitochondrial construction and delivery and provide solid experimental support for the use of this strategy for the clinical treatment of bone metabolic diseases.

### 5.3. Bone-Targeted Nanodelivery

Bone-targeted nanodelivery refers to the use of the size effect and surface modification characteristics of nanomaterials, combined with the specific recognition sites of bone tissue and bone-related cells (such as bone marrow mesenchymal stem cells and osteoblasts), to construct nanodelivery carriers for delivering drugs, bioactive molecules or organelles (such as mitochondria) to bone tissue, osteocytes and targeted subcellular structures. New delivery technologies are important for the precise treatment of bone metabolic diseases. Sun et al. designed and constructed functionalized Fe_3_O_4_ nanoparticles (KGM-PEG-SPIONs) for use as a bone-targeted nanodelivery system guided by the regulation of mitochondrial transport. The nanoplatform was found to enhance the mitochondrial quality of macrophages by activating autophagy, promoting the biosynthesis of iron–sulfur clusters, increasing the polarization of M2 macrophages, and enhancing their compatibility with the oxidative and inflammatory microenvironment of senescent BMSCs. These M2-like mitochondria were transferred to senescent bone marrow mesenchymal stem cells through CX43 gap junctions to restore their membrane potential, ATP production ability, calcium homeostasis and osteogenic differentiation [112]. Zhang et al. designed and constructed a cerium-based nanosystem (CNS). This nanosystem effectively restored the survival rate and bone formation function of senescent bone marrow mesenchymal stem cells (S-BMSCs) by promoting their mitochondrial biogenesis and the metastasis of senescent bone marrow-derived macrophages (S-BMDMs). Mechanistically, the CNS effectively alleviated the accumulation of ROSs in S-BMDMs through autophagy activation, restored the survival ability of S-BMDMs, and promoted their polarization to M2 macrophages. In addition, CNS-activated autophagy restored NAD^+^ levels in S-BMDMs, thereby reactivating SIRT1. Activation of this cascade subsequently triggered the SIRT1-PGC-1α-Nrf2-TFAM axis to drive mitochondrial biosynthesis in S-BMDMs. Moreover, the CNS promoted the transfer of mitochondria from S-BMDMs to S-BMSCs by promoting TNT-mediated mitochondrial transfer, thereby enhancing osteogenic differentiation and inhibiting adipogenesis [147].

Studies of the above two nanosystems have not only explored the construction and regulatory mechanism of bone-targeted nanodelivery systems in terms of mitochondrial transport but also provided new nanodelivery strategies for precise intervention in osteoporosis. These studies have also provided an important theoretical and experimental foundation for the preclinical translation of bone-targeted nanodelivery systems mediated by mitochondrial transport, thereby advancing the field. However, studies on this topic are rare. In the future, more attention should be given to bone-targeted nanodelivery systems to provide new perspectives for clinical treatment.

The biocompatibility of nanoparticles and the potential to elicit immune responses remain key issues for the use of nanosystems in mitochondrial transfer. Nanoparticles have been shown to impair mitochondrial function in target cells and affect key mitochondrial signaling pathways, including those involved in apoptosis and autophagy [148,149]. One of the most serious challenges limiting the success of nanoparticles in clinical applications is reaching the treatment site in sufficient concentrations without accumulation in nontarget tissues.

## 6. Summary and Future Prospects

This review comprehensively explores the molecular mechanism of mitochondrial transport-mediated regulation of bone metabolism homeostasis and targeted intervention strategies in bone metabolic diseases. It focuses on the key molecules and signaling pathways involved in the regulation of bone homeostasis and bone metabolism, the pathological mechanism of mitochondrial transport abnormalities involved in the occurrence and development of bone metabolic diseases, and the research status and translational prospects of targeted mitochondrial transport in the treatment of bone metabolic diseases. In recent years, many studies have confirmed that mitochondrial transport-related pathways and key molecules play important roles in the regulation of osteoblast and osteoclast function and the process of bone metabolic disease. On this theoretical basis, researchers have investigated the treatment of bone metabolic diseases by targeting mitochondrial transport and have made some progress, such as targeting key molecules involved in mitochondrial transport, mitochondrial transplantation and stem cell-mediated mitochondrial delivery and repair, providing new ideas for the treatment of bone metabolic diseases. However, many bottlenecks still exist in this field. First, the molecular mechanism through which mitochondrial transport regulates bone metabolism homeostasis has not been fully elucidated. The existing research has focused on single molecules or signaling pathways, and the understanding of multimolecular synergy and cross-cell regulatory networks is still not comprehensive. Second, for bone metabolic diseases mediated by abnormal mitochondrial transport, specific and effective targeted therapies are lacking, and many bottlenecks in their clinical application still exist, making it difficult to meet the needs of clinical diagnosis and treatment.

Future research can focus on comprehensively analyzing the molecular mechanism of mitochondrial transport involved in the regulation of bone metabolism homeostasis through molecular tracking and cell sequencing; on the basis of the relevant research results, more small-molecule compounds targeting mitochondrial transport core proteins should be explored to clarify their mechanism of action in different bone metabolic diseases, and the structure of the compounds should be optimized to improve their targeting and bioavailability. Moreover, molecular-level drug screening techniques such as high-throughput screening and virtual screening have been used to assist in the development of targeted drugs. The long-term mechanism through which mitochondrial transplantation exerts its effects should be explored. Using single cell sequencing, molecular tracking and other technologies, the survival, proliferation and function of transplanted mitochondria in target cells can be tracked, and the long-term molecular pathways regulating bone metabolism homeostasis can be clarified. Understanding the mechanisms that may lead to potential adverse reactions will provide theoretical support for the optimization of treatment options. Research should focus on technical breakthroughs in the targeted delivery of biomaterials, optimizing the composition and structure of biomaterials (such as 3D bionic hydrogels), improving their biocompatibility, targeting and mitochondrial loading capacity, developing efficient large-scale preparation processes, and reducing production costs. Moreover, bone-targeted nanodelivery technologies and efficient and accurate advanced mitochondrial delivery systems are needed. Future studies should optimize the mitochondrial delivery efficiency, stability and safety, comprehensively verify the survival status, functional integrity and long-term effects of mitochondria in target cells after transplantation, and further improve the whole process of mitochondrial construction and delivery to break through the clinical translational bottlenecks. These studies will provide new ideas and directions for the discovery of additional therapeutic targets for bone metabolic diseases and the development of new drugs. We believe that further research on the molecular mechanism through which mitochondrial transport regulates bone metabolism and the continuous optimization of targeted intervention technologies will provide more solid theoretical support and more effective clinical strategies for the treatment of bone metabolic diseases, thereby advancing the research field of bone metabolic diseases.

## Data Availability

No new data were created or analyzed in this study. Data sharing is not applicable to this article.
